# Whole-genome sequencing of esophageal adenocarcinoma in Chinese patients reveals distinct mutational signatures and genomic alterations

**DOI:** 10.1038/s42003-018-0182-8

**Published:** 2018-10-24

**Authors:** James Y. Dai, Xiaoyu Wang, Matthew F. Buas, Chengjuan Zhang, Jie Ma, Bing Wei, Yin Li, Baosheng Zhao, Teresa S. Hyun, Xueyan Chen, Keith R. Loeb, Robert Odze, Lena Yao, Xin Sun, Steve Self, Thomas L. Vaughan, Yongjun Guo

**Affiliations:** 1 Public Health Sciences Division, Fred Hutchinson Cancer Research Center, Seattle, 98109 WA USA; 2 Department of Biostatistics, University of Washington, Seattle, 98195 WA USA; 3 Department of Cancer Prevention and Control, Roswell Park Cancer Institute, Buffalo, 14203 NY USA; 4 Department of Molecular Pathology, The Affiliated Cancer Hospital of Zhengzhou University, Zhengzhou, 450008 He Nan Province China; 5 Department of Thoracic Surgery, The Affiliated Cancer Hospital of Zhengzhou University, Zhengzhou, 450008 He Nan Province China; 6 Department of Thoracic Surgery, The First Affiliated Hospital of Xinxiang Medical College, Xinxiang, 450008 He Nan Province China; 7 Clinical Research Division, Fred Hutchinson Cancer Research Center, Seattle, 98109 WA USA; 8 Department of Pathology, University of Washington, Seattle, 98195 WA USA; 9 Department of Laboratory Medicine, University of Washington, Seattle, 98195 WA USA; 10 Department of Pathology, Brigham and Women’s Hospital, Boston, 02115 MA USA; 11 Vaccine and Infectious Disease Division, Fred Hutchinson Cancer Research Center, Seattle, 98109 WA USA; 12 Institute of Occupational Health and Poison Control, Chinese Center for Disease Control and Prevention, Beijing, 100050 China; 13 Department of Epidemiology, University of Washington, Seattle, 98195 WA USA

## Abstract

While the incidence of esophageal adenocarcinoma (EAC) has risen drastically in Western countries over the last 40 years, a similar trend has not been observed for EAC in China. Here, we analyzed mutational spectrum, copy number alterations, and structural variants from whole-genome sequencing of 10 Chinese EAC tumor samples and their matched normal samples, and compared them to previously reported EAC tumor specimens from Western countries. The mutational burden in Chinese EAC was significantly lower than that found in EAC from Western countries. The hallmark A>C mutational signature observed at high frequency in EAC from Western countries, which has been linked to acid reflux, is completely absent in Chinese samples. Furthermore, none of the Chinese samples showed evidence of chromothripsis and genome doubling that are often found in EAC from Western countries. In summary, Chinese EAC tumor samples had distinct genomic profiles and signatures, suggesting that EAC in Chinese individuals may arise from a different etiological pathway.

## Abstract (web summary)

James Dai et al. report the first whole-genome sequencing study of esophageal adenocarcinoma (EAC) in Chinese patients. They find distinct genomic alterations and signatures compared to EAC in patients from Western countries.

## Introduction

Esophageal cancer is the eighth most common cancer worldwide and the sixth leading cause of cancer-related mortality^1^. There are two main histological subtypes, esophageal squamous cell carcinoma (ESCC) and esophageal adenocarcinoma (EAC), the incidences of which differ by geographic regions. In Western countries, EAC has undergone a sharp rise in incidence since the early 1970s^2^ and has surpassed ESCC as the most common histologic type; established risk factors include gastroesophageal reflux disease (GERD), obesity, and smoking^3–5^. In East Asian countries, no obvious increase in the incidence of EAC has been observed^6–11^, and the most dominant histological type remains to be ESCC in many countries including China; risk factors for both subtypes are less well established.

Most reported cases of EAC in Western countries are believed to arise from Barrett’s esophagus, an epithelial precursor lesion of the esophagus characterized by replacement of the native squamous epithelium with columnar epithelium, as an adaptive response to chronic gastric acid reflux^12^. Only a small fraction of Barrett’s esophagus progresses to dysplasia and leads to the development of EAC. In a recent review, the prevalence of GERD in China was reported to be the lowest among countries with data available, around 2.5–5% depending on the specific study^13–15^. Barrett’s esophagus is rather rare in China and has not been well studied^16^. In a recent retrospective case report on esophageal cancers in Henan Cancer Hospital, China, we have found that Barrett’s esophagus was rarely associated with EAC (4.6%, 10 out 217 EAC identified in 2002–2011), much lower than the reported detection rates around 80–90% in Western EAC^17^, raising the possibility that Chinese EAC may arise via a different etiological pathway from US EAC.

A number of large-scale, whole-genome, and whole-exome sequencing studies have been conducted in the US and UK to investigate the genomic landscape of EAC, identifying molecular alterations and genomic signatures and providing additional insight into cancer etiology^18–22^. Briefly, while there is widespread chromosomal instability and high mutational load in the EAC genome, presumably due in part to chronic inflammation and oxidative stress in response to acid reflux, only a limited number of recurrent tumor driver genes have been identified, including *TP53*, *SMAD4*, *ARID1A*, and *CDKN2A*. A mutational signature represented by a high A>C transversion rate at AA sites has been consistently identified and potentially linked to acid reflux with some preliminary evidence^18–20^. Other than this dominant molecular subtype, mutational spectrum analysis suggests that there are several less frequent subtypes including a BRCA signature, with potential therapeutic relevance^19^. Chromothripsis, a genomic catastrophic event characterized by tens to hundreds of locally clustered DNA rearrangements, has been found to be frequent in EAC^20^, which may have an important role in the malignant transformation of EAC.

Because Chinese EAC appears to be less associated with GERD and Barrett’s esophagus, it is of interest to characterize the Chinese EAC genome in comparison to the EAC genome from Western countries, though there has not been any genome-wide interrogation of Chinese EAC. Here, we describe the landscape and the spectrum of genomic alterations in 10 fresh-frozen, surgically resected tumor samples from Chinese EAC patients in Henan Cancer Hospital, China. These tumors and matched normal samples were subjected to whole-genome sequencing (WGS) and profiling for genome-wide mutations, copy number alterations, and structural changes. To minimize the potential impact of analysis pipelines, we systematically compared these Chinese EAC genomes to previously published WGS data from the 16 US EAC samples previously reported^18^ and sequenced to a similar depth, using identical bioinformatics procedures. In addition, we have also downloaded mutational data from the 301 UK EAC samples^19^ from ICGC and added these data in the comparison. Our hypothesis is that Chinese EAC presents fewer mutations and chromosomal rearrangements because the usual association between Barrett’s esophagus and EAC in surgical samples is absent in this population of Chinese EAC^17^. The results suggest that Chinese EAC indeed has very different genomic characteristics relative to EAC from Western countries, namely less mutations, a low A>C transversion rate, and no evidence of chromothripsis and genome doubling.

## Results

### Patient and tumor characteristics

The patient and tumor characteristics for 10 Chinese EAC samples being sequenced are shown in Supplementary Table 1. The mean age of these 10 patients is 58.3 years (minimum 45, maximum 75), and six of the 10 are male. Consistent with our previous larger study of 217 Chinese EAC tumors, only three of the 10 tumors were located in the lower third of the esophagus. The remaining seven were located in the middle thoracic esophagus, in contrast to the observation that the majority of EAC tumors in Western countries today arise from the lower esophagus. Note that historically before the rise of the EAC incidence in the US in the 1970s, the rate of EAC in the lower esophagus appears to be close to the rate of EAC in the middle esophagus, which remained stable since, and the rise of EAC incidence was driven by the lower esophagus. (EAC incidence data, 1973–2014, from SEER9 registry, Supplementary Figure 1.)

### Landscape of mutations in Chinese EAC

**Fig. 1:**
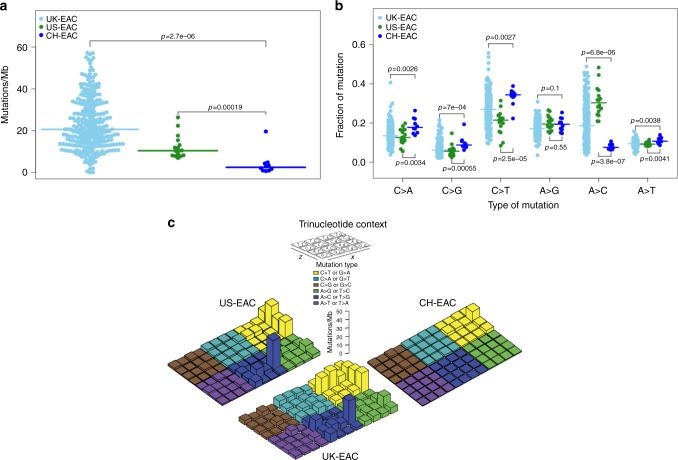
Comparisons of mutation burden and types based on whole-genome sequencing data from Chinese EAC, US and UK EAC. **a** The number of mutations per million base pairs. **b** The proportion of six types of mutations. **c** Mutation burden of six mutation types in the trinucleotide context

We performed WGS on tumor-normal pairs from 10 Chinese EAC cases. DNA samples from both tumors and matched adjacent normal tissue samples were sequenced to 30× coverage with paired 150-bp reads using Illumina HiSeq 2500 instruments. We identified a median of 7048 mutations per tumor genome for Chinese EAC (range: 2070–53,570), corresponding to a median mutation frequency of 2.56 mutations/Mb (range: 0.75–19.65). The median mutation frequency was higher in intergenic regions (3.45 mutations/Mb), intermediate in intronic regions (2.12 mutations/Mb), and lowest in coding exons (2.08 mutations/Mb). Compared to 9.9 mutations/Mb previously reported and replicated by us in the 16 US EAC^18^, the mutation frequencies in these Chinese EAC tumors were significantly lower (Fig. 1a, Wilcoxon rank sum test, *p*-value = 1.9e−4). The median density of nonsilent mutations (including missense, nonsense, splice site, frameshift, and in-frame indel) was 0.92 mutations/Mb (range: 0.09–13.56), also significantly lower than that observed in the 16 US EAC samples (median 4.18, range 2.95–8.72, Wilcoxon rank sum test, *p*-value = 3.2e−4). Similarly, the mutation burden in the 301 UK EAC is significantly higher than that observed in the Chinese EAC (Fig. 1a, median 20.64, range 0.12–57.19, Wilcoxon rank sum test, *p*-value = 2.7e−6 when compared to Chinese EAC). Comparing to US EAC, UK EAC have a slightly higher mutation burden because it included more severe EAC samples (for example, Chinese EAC and US EAC have the same median of tumor stage 2, while the median is 3 for UK EAC).

Kataegis mutation clusters results are shown in Supplementary Figures 2 and 3. The detected kataegis is listed in Supplementary Table 2. Five kataegis foci were detected in 3 Chinese EAC samples, and 47 kataegis were detected in 12 US EAC samples. Compared to Chinese samples, US EAC samples tend to have more kataegis (Wilcoxon rank sum test, *p*-value = 0.019).

### Mutational signatures in Chinese EAC

We analyzed the spectrum of mutation types in Chinese EAC versus US and UK EAC. A notable difference between Chinese EAC and US EAC was that Chinese EAC had far fewer A>C transversions (Fig. 1b, median percentage 7.51%, range 6.27–10.62%, Wilcoxon rank sum test, *p*-value = 3.77e−7 when compared to US EAC), the mutational signature consistently shown to be associated with US/UK EAC. In stark contrast, the median percentage of A>C transversions in US EAC was reported to be 30.30%. Among the six mutation types, C>T transitions have the highest frequency (median 34.37%, range 22.31–38.87%), which has been seen in most epithelial cancers including US EAC. When comparing the proportions of six possible base changes, five of them show significant differences between US EAC and Chinese EAC (Fig. 1b), including A>C, A>T, C>A, C>G, and C>T. After placing single base changes into surrounding trinucleotide contexts (Fig. 1c), we found that in Chinese EAC, a median percentage of 31.16% of A>C mutations were flanked by a 5′ adenine, in contrast to 70.44% in US EAC. Overall, A>C transversions at AA sites accounted for 2.40% of total mutations in Chinese EAC, which is 10-fold lower than the proportion of A>C transversions at AA sites in US EAC (Fig. 1c, 22.21%, Wilcoxon rank sum test *p*-value = 3.77e−7). When we compared Chinese EAC relative to the UK EAC, the results are similar that Chinese EAC have a lower A>C transversion rate (Fig. 1b, c). There is no material difference in mutational signatures between US EAC and UK EAC.

**Fig. 2:**
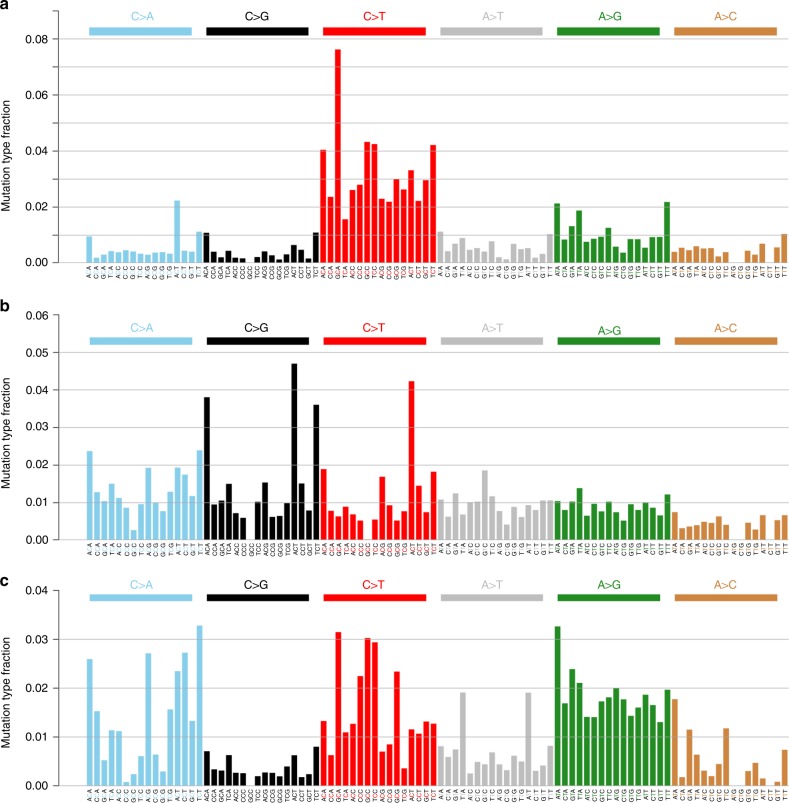
Mutational signatures in Chinese EAC genomes discovered by the non-negative matrix factorization algorithm^23^. **a** A S1-like signature  with C>T in a *CG context, which may be associated with aging processes. **b** A S3-like signature with more-or-less equal representation of all 96 tri-nucleotide mutation types, possibly linked to defects in BRCA1/2-led homologous recombination pathway. **c** The third signature which appears to be an unknown one, likely a mix of S1 (similarity = 0.71) and U2 (similarity of 0.80), an unvalidated mutation signature defined by Alexandrov et al.^23^

We performed the mutational signature analysis in a three-base context via a non-negative matrix factorization (NMF) algorithm^23^. We obtained the signature stability and reconstruction error using the NMF algorithm^23^. Shown in Supplementary Figure 4, three signatures give the optimal trade-off between signature stability and reconstruction error and were thus selected as the more likely configuration that may explain the observed data. We used the cosine similarity to assign them to those signatures defined by Alexandrov et al.^23^. Two signatures previously described in EAC in the UK^19^ were also discovered in Chinese EAC (Fig. 2): S3 (27% of samples), a signature with more-or-less equal representation of all 96 tri-nucleotide mutation types, linked to defects in BRCA1/2-led homologous recombination pathway; and S1 (49% of samples), a signature with C>T in a *CG context, likely associated with aging processes. These mutational signatures represented a minority of several subgroup signatures identified in EAC data from the UK^19^. The hallmark signature for EAC from the US and UK (S17), that is marked predominantly by A>C substitutions, is completely absent in our analysis of Chinese EAC tumors. The last signature seems to be an unknown one, or a mix of S1 (similarity = 0.71) and U2 (similarity of 0.80), an unvalidated mutation signature defined by Alexandrov et al.^23^. We further conducted signature analysis using *deconstructSigs* ^55^, and the example results are shown in Supplementary Figure 5. We set the maximum number of signatures as three (signatures.limit = 3) and the signature cutoff as 0.15 (signature.cutoff = 0.15) to obtain major signatures for each sample. We detected the same set of signatures (S1, S3, and U2) among those individual samples, which confirmed the results shown in Fig. 2. To complete the comparison, we conducted the signature analysis on 16 US samples with the similar approach and we detected four signatures, S1 (38% of samples), S3 (19% of samples), S17 (17% of samples), and an unknown signature (mix of S1 and U2, 25% of samples). These results are shown in Supplementary Figure 6.

### Genes mutated in Chinese EAC

We observed non-silent mutations in 691 genes (including missense, splice site, frameshift, nonsense, in-frame indel), of which 22 were mutated in two or more patients (Supplementary Figure 7). These high-abundance mutated genes include *NBPF20*, *NOTCH1*, *TP53*, *MUC16*, *F8*, *HARS*, and *TTN*. Most of them could be false-positive findings due to their long coding regions, such as *TTN* and *MUC16*. To identify genes showing evidence of positive selection for mutation, we used the mutation significance algorithm MutSigCV2.0^24^. This tool compares the mutation occurrence in each gene to the background mutation rate. *TP53* was found to have a *p*-value less than 0.05 and a mutation frequency greater than 20%. However, neither one was identified as a significantly mutated gene after correction for multiple comparisons (*q*-value < 0.1), likely due to the small sample size for Chinese EAC (*n* = 10).

We performed the gene set enrichment analysis on the mutated genes using Enrichr^25^. Among the highly frequently mutated genes (genes mutated in at least two samples), *NOTHC1* and *TP53* were enriched in thyroid hormone (TH) signaling pathway. The link of TH signaling to the development of esophageal cancer has been previously reported^26^. Among all the mutated genes (genes mutated in at least one sample), we found *COL17A1, SLC8A3, COL14A1, COL5A3, COL6A2, COL12A1, COL4A6, ATP1B2* were enriched in protein digestion and absorption pathway. This pathway was also identified in a previous esophageal cancer study^27^. Furthermore, *ATF2, CHUNK, PKN3, NOS3, LAMA1, CSH1, LAMA3, TSC1, PPP2R2A, TNN, CREB3L1, COL6A2, CHAD, DD1T4, KDR, COL4A6, BCL2, PKN2, TP53, and TLR4* were enriched in PI3K-Akt signaling pathway. This pathway plays an important role in tumor genesis and resistance, and it was investigated in a previous EAC study^28^.

### Somatic copy number alterations (SCNA) in Chinese EAC

**Fig. 3:**
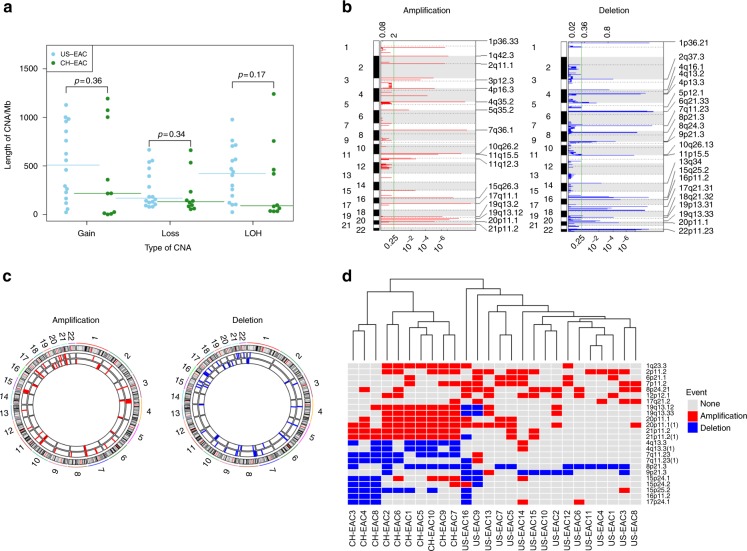
Comparisons of somatic copy number alterations (SCNA) between Chinese EAC and US EAC. **a** The length of SCNAs by copy gain, copy loss, and loss of heterozygosity (LOH). **b** Recurrent focal SCNAs in Chinese EAC detected by GISTIC 2.0. **c** The CIRCOS plot comparing recurrent SCNAs between US EAC and Chinese EAC. The inner circle represents Chinese EAC and the outer circle represents US EAC. **d** Unsupervised hierarchical clustering using recurrent SCNA among 10 Chinese EAC samples and 16 US EAC samples

We used Control-FREEC to normalize and segment copy number data based on the copy ratio between tumor samples and matched normal tissue samples^29^. The tumor ploidy and percentage of tumor content were estimated by the ABSOLUTE algorithm^30^ and shown in Supplementary Table 3. Like US EAC, substantial somatic copy number alterations (SCNA) were observed in the Chinese EAC genome, including copy number gain, loss, and loss of heterozygosity (LOH), on average occurring in 29.7% genome. As shown in Fig. 3a, the median lengths of SCNAs in Chinese EAC were 218.2 Mb for copy gain, 133.0 Mb for copy loss, 90.9 Mb for LOH. These median numbers are all slightly smaller than those in US EAC, however the differences of these global measures of copy number alterations are not significant (Fig. 3a, *p*-value = 0.363, 0.336, 0.165, respectively).

The segmented SCNAs were analyzed by GISTIC 2.0 to define recurrently amplified and deleted regions^31^ (Fig. 3b). There were major differences between US and Chinese EAC in recurrent SCNAs as identified by GISTIC. On the arm-level, we found amplifications on 8q, 10p, 20p, and 20q (with GISTIC *q*-value < 0.1) in US EAC, while we identified a deletion on 21p (with GISTIC *q*-value < 0.1) in Chinese EAC. On the focal level, 17 recurrent copy number gains were detected in Chinese EAC, covering 44.9 Mb of the cancer genome, and 22 recurrent gains were found in US EAC, covering 81.3 Mb of the cancer genome. The number of chromosomal regions with recurrent copy number losses in Chinese EAC was 17, covering 25.1 Mb of genome, which was lower than the number of recurrent losses in US EAC (22, covering 44.9 Mb). The recurrent regions and genes are listed in Supplementary Data 1.

The recurrent amplification SCNAs in Chinese EAC (but absent in US EAC) included the following regions: 1q42.3, containing *AKT3*, a regulator of cell signaling in response to growth factors and a contributor to the aggressiveness of steroid hormone-insensitive carcinomas^32,33^; 11p15.5, containing (i) *MUC6*, a mucin thought to play a major role in the protection of the gastrointestinal tract from acid^34^, (ii) *SCT*, a glucagon-family prohormone shown to inhibit gastric acid secretion^35^, and (iii) *HRAS*, an oncogene linked to gastric carcinoma cell aggressiveness^36^; and 15q26.3, containing *IGF1R*, which has been documented in various malignancies of the gastrointestinal tract, such as colorectal and pancreatic carcinomas^37^.

Distinct deletion SCNAs for Chinese EAC include the following regions: 1p36.21, containing (i) *MTOR*, a central regulatory kinase dysregulated in gastric cancer and activated by *PI3K/Akt* (including aforementioned *AKT-3*) and insulin-like growth factor receptor (including aforementioned *IGF1R*)^38^, and (ii) *CHD5*, a tumor suppressor gene in gastric cancer^39^. The recurrent deletion SCNAs for US EAC (but absent for Chinese EAC) include 16q23.1, which contains a tumor suppressor gene *WWOX*, whose deregulation is associated with gastric cancer^40^ and ESCC^41^; and 18q21.2, which contains *SMAD4*, a tumor suppressor gene associated with gastrointestinal carcinogenesis^42^.

To visualize the overlapping regions of SCNA for Chinese and US EAC, CIRCOS^43^ plots of recurrent SCNAs were generated (Fig. 3c). Only six recurrently amplified regions were found in both US EAC and Chinese EAC: 3p12.3, 4p16.3, 7q36.1, 10q26.2, 17q11.1, and 21p11.2, covering 13.8% of all amplified regions. Similarly, only four shared deleted regions were identified: 5q12.1, 6p22.1, 8p21.3, and 9p21.3, covering only 6.7% of all deleted regions. To investigate whether the recurrent regions can distinguish Chinese EAC and US EAC, we combined the two datasets and performed a joint GISTIC analysis to obtain a set of 13 focal amplification and 11 deletion regions. This set of SCNAs was used as features in an unsupervised hierarchical clustering algorithm, resulting in complete separation of Chinese EAC from US EAC (Fig. 3d). Taken collectively, these results suggest that Chinese EAC and US EAC have distinct SCNA profiles.

Because the mutational signature S3—defects in BRCA1/2-led homologous recombination pathway—was detected in Chinese EAC, we examined copy number alterations in DNA repair genes. Among the 41 genes involved in homologous recombination pathway defined by KEGG, we identified copy number deletions in eight genes: *ATM*, *BLM*, *BRIP1*, *RAD50*, *RBBP8*, *UIMC1*, *RAP2*, and *RAD54L*, among the 5 samples with the S3 signature detected by *deconstructSigs*^55^, which corroborates evidence of the mutational signature S3 in the Chinese EAC.

### Structural variants in Chinese EAC

**Fig. 4:**
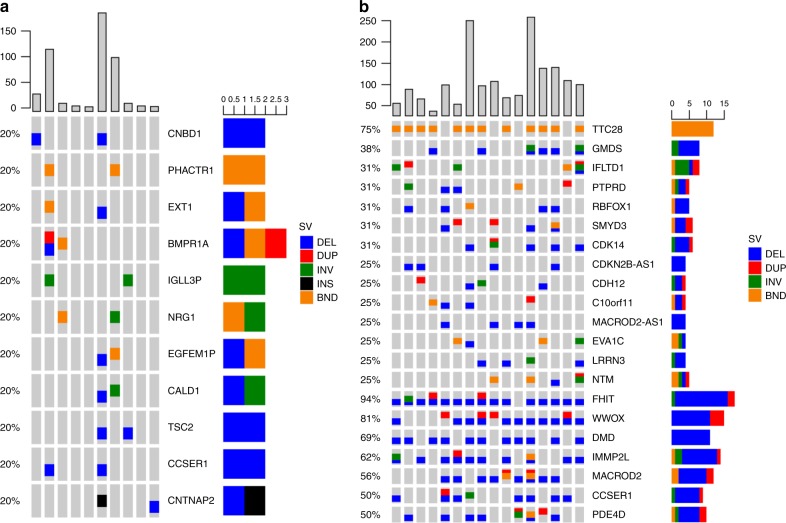
Recurrent structural variants detected by Manta^44^ for Chinese EAC (**a**) and US EAC (**b**). The bar on the top shows the total number of structural variants in the genome and the bar on the right shows the number of samples having the variant

We also analyzed the WGS data using Manta^44^ to identify structural variants (SVs). A total of 795 candidate variants were identified, with a median of 21 per tumor (range: 8–385), far fewer than observed in the 16 US EAC genomes (median: 172, range: 77–452). We then mapped these SVs at the gene level and searched for recurrently rearranged genes. No genes were found to be rearranged in more than 20% of samples in Chinese EAC genomes. Eleven genes were found in 2 samples (Fig. 4a), including *TSC2*, a regulatory gene involved in the *MTOR* signaling pathway^45^; *BMPR1A*, a receptor serine/threonine kinase, deletion of which may be associated with Juvenile polyposis syndrome, a rare autosomal dominant disorder characterized by multiple gastrointestinal juvenile polyps and an increased risk of colorectal cancer^46^; and two genes, *CCSER1* and *CNTNAP2* (on the last two rows of Fig. 4a), residing in fragile sites^47^. By contrast, in 16 US EAC genomes, we observed several recurrently rearranged genes (Fig. 4b): translocation of *TTC28* in 75% samples, which was the most frequent translocation in colon and rectal cancer^48^; deletion of *GMDS* in 37.5% samples, which was also found in gastric cancer^49^; and alterations of *RBFOX1*, *SMYD3*, or *CDK14* in 31% of samples, which were also found in EAC from UK^19^. Furthermore, seven fragile genes (on the bottom of Fig. 4b) were found in more than 50% of samples in US EAC genomes, including *FHIT* (94%), *WWOX* (81%), *DMD* (69%), *IMMP2L* (63%), *MACROD2* (56%), and *CCSER1* (50%), most of which (except DMD) were also found in UK EAC^19^.

Chromothripsis, a one-step catastrophic genomic event characterized by local concentration of tens to hundreds of SVs in one or few chromosomes, has been reported to be frequent (32%) in EAC from Western countries^20^. Such one-step catastrophic structural rearrangement was thought as an alternative driving force of cancer development and progression in addition to the stepwise accumulation of mutations^48^. Based on a set of criteria previously used to identify chromothripsis, we verified that 5 out 16 (31%) US EAC samples contain rearrangement similar to chromothripsis (Supplementary Figure 8), while none of the Chinese EAC samples showed evidence of chromothripsis (Fisher's exact test *p*-value = 0.03, comparing 0% in 10 Chinese EAC samples to 32% previously reported in 123 EAC from Western countries). There is no evidence of genome doubling events in Chinese EAC.

The CIRCOS plots for Break–Fusion–Bridge (BFB) identified in Chinese EAC and US EAC are shown in Supplementary Figure 9. We identified 6 BFBs out of 16 US EAC samples and 1 BFB out of 10 Chinese EAC samples. Although the proportion of BFB in US samples is higher, the difference is not significant due to the limited sample size (Fisher's exact test *p*-value = 0.19)

## Discussion

In this article, we described for the first time to our knowledge the landscape of genomic alterations in Chinese EAC in comparison to EAC sequencing data in Western countries. This set of Chinese EAC samples was obtained from a tumor repository in a Chinese hospital. Using case series over 10 years in this hospital, we have previously reported that the rate of Chinese EAC has not been increasing and BE is rarely detected in surgical samples from Chinese EAC patients^17^. In Western countries, genomic features of EAC have been well studied: high mutation burden, high chromosomal instability, and a unique A>C signature, all of which may reflect, in part, exposure of the lower esophageal epithelium to a harsh, genotoxic environment created by acid reflux. Three lines of preliminary evidence from this genomic analysis support the hypothesis that Chinese EAC arises from a different etiological pathway. First, the hallmark mutational signature (high A>C mutation rate) of Western EAC, which has speculatively been linked to acid reflux, was completely absent in Chinese EAC samples. Second, the overall mutational burden in Chinese EAC (median 2.56 mutations/Mb) was significantly lower than that observed in US EAC (median 9.9 mutations/Mb, Wilcoxon rank sum test *p*-value = 1.9e−4), which is also consistent with the evidence that the high A>C (or T>G) mutation rate is associated with a high mutational load and neoantigen burden^19^. Third, the degree of chromosome instability and copy number alterations in Chinese EAC was less than that observed and reported in US EAC, and there were few overlaps in recurrent SCNAs. Intriguingly, these genomic differences are mirrored by differences in the predominant anatomic location of EAC tumors in the US versus Chinese patients; as described previously, in contrast to US EACs, which typically arise in the reflux-exposed lower third of the esophagus (~80%), Chinese EACs appear more likely to be found in the middle third of the esophagus (~65%), with only 32% arising in the lower third^17^. Of interest, exploratory analysis of US cancer registry data (SEER) suggests that in the early 1970s, a smaller percentage of US EACs arose in the lower third of the esophagus (45%+), with more tumors arising in the middle third than observed subsequently, as overall incidence continued to rise (Supplementary Figure 1). It remains to be seen whether the incidence and anatomic distribution of Chinese EACs will shift towards US patterns with continued Westernization trends.

A recent analysis of WGS data from 129 EAC cases from the UK suggests that EAC is a heterogeneous disease, which may be composed of three distinct molecular subtypes: (i) the dominant A>C mutational pattern, (ii) a C>A/T mutational pattern with evidence of an aging imprint, and (iii) enrichment for BRCA signature^19^. Our data in 10 Chinese EAC cases reveal patterns of the two less frequent EAC subtypes in the UK data: high C>A/T mutations and some enrichment for BRCA signatures (Fig. 2). This intriguing finding raises the possibility that tumor subtypes comprising a minority of EAC cases in Western countries, i.e., those potentially arising from aging and defects of DNA damage repair, may constitute the majority of EAC cases in China. As elaborated previously, the sequencing-based subgroups have important implications for developing novel therapeutics and selecting effective treatments.

We did not detect any genome doubling event in the 10 Chinese EAC samples, which is consistent with a lower rate of chromothripsis and BFB in Chinese EAC. It seems that comparing to US EAC, Chinese EAC lacks these global SVs.

As been reported in the UK study^19^, we also identified several genes (such as *HRAS*, *AKT3*, *FGFR3*, *NF1*, *INS*, *IGF1*, *HGF*, *IGF1R*, and *GNG4*) amplified in the MAPK/ERK and PI3K pathways. We thus observed widespread gene amplification across multiple receptor tyrosine kinases (RTKs) as well as downstream within the MAPK and PI3K pathways, which suggested potential resistance mechanism against RTK targeted therapy could be co-existing amplifications of genes located downstream of RTK.

We also investigated the comparison of Chinese EAC and Chinese ESCC. We studied 17 WGS samples from Song et al.'s ESCC study^50^. Generally, Chinese EAC and ESCC have different genomic features. We checked its mutation burden and mutation subtypes. The comparison of ESCC and our Chinese EAC is shown in Supplementary Figure 10. Comparing to US EAC, they have lower mutation burdens and similar fractions of mutation subtypes. Comparing to ESCC, Chinese EAC has lower mutation burden and lower “C>G” mutational fraction. Mutational signatures of ESCC were studied by Zhang et al.^51^. They identified three signatures: S2 (attributed to the activity of the AID/APOBEC family of cytidine deaminases), S1 (aging), and an unknown one. It seems APOBEC-catalyzed deamination is the main source of DNA damage in ESCC while in Chinese EAC, instead of S2, we found the signature S3 (was also found in UK EAC), which is related to the failure of DNA damage repair. As for SCNV, we compared our Chinese EAC result with ESCC from TCGA (Integrated genomic characterization of oesophageal carcinoma); we found substantial differences in patterns of alterations between Chinese EAC and ESCC. There are only 2 common amplified regions (19p13.2 and 19q13.12), 2 common deletion regions (9p21.3 and 19p13.3) identified in both datasets.

There are several limitations in our study. First, our restricted sample size (10 pairs of tumors and matched normal samples) for WGS did not allow for the detection of recurrent cancer genes and alterations with low recurrent frequencies (e.g., less than 20%). Second, our sequencing depth of 30× on average was adequate for assessing chromosomal instability, but potentially so less for discovering minor somatic mutations and genes particularly for highly heterogeneous tumor samples. For example, we are not able to discover any significantly mutated genes using MutSigCV2.0 with a *q*-value less than 0.05. Third, the samples were extracted from a tumor repository in a Chinese hospital with only limited information on potential risk factors such as acid reflux symptoms, obesity, and smoking. We are not able to link these newly discovered genomic alterations to environmental exposures and risk factors, to aid in interpretation.

In closing, our analysis has revealed a number of distinctive genomic differences between Chinese and US EAC tumors. These results underscore the need for additional integrative, comparative etiologic studies of this cancer in Western and East Asian countries. Such work has the potential to further elucidate causes of the Western EAC epidemic, while at the same time, to inform tailored preventive, diagnostic, and therapeutic strategies across geographically-disparate at-risk populations.

## Methods

### Patients and sample collection

EAC patients were identified and recruited for study participation in Henan Cancer Hospital between 2009 and 2015. Samples were obtained from patients who had received no previous chemotherapy or radiotherapy for their disease. Each frozen primary tumor specimen had an adjacent non-tumorous esophageal tissue. Pathology quality control was performed by a group of pathologists on each tumor and adjacent normal tissue specimen from a frozen section slide to confirm that the tumor specimen was histologically consistent with EAC, according to the guidelines in the American Joint Committee on Cancer (7th edition), and that the adjacent tissue specimen contained no tumor cells. This study was approved by the Institutional Ethics Committee of Henan Cancer Hospital. Tumor samples with confirmed pathology, >75% tumor nuclei and <20% necrosis were submitted for nucleic acid extraction.

### Whole-genome sequencing data processing and quality control

DNA was extracted using pheno-chloroform extraction. WGS was conducted at Beijing Genomics Institute, Shenzhen, China. DNA samples were sequenced to 30× on average using Illumina HiSeq 2500 instruments, and 150-bp paired-end reads were obtained. The processing and analysis of WGS data were performed using Broad Institute pipelines, following Genome Analysis Toolkit (GATK) Best Practices^52^. Paired-end reads were mapped to the human reference genome (hg19) using multi-threaded BWA^53^ and a BAM file was generated for each tumor and matched normal sample. To assess the alignment quality, the FastQC package was used (http://www.bioinformatics.babraham.ac.uk/projects/fastqc), and GATK ConEst^52^ was used to check cross-contamination between tumor samples. To compare WGS data from China to data from the US, we used WGS data from the 16 EAC cases^18^ stored in the database of Genotypes and Phenotypes (dbGaP) (study accession: phs000598.v1.p1).

### Variant calling methods

Somatic mutations were called using MutTect (version 1.1.7) and indels were called by GATK IndelGenotyper^52,54^. Functional annotation of mutations was performed with Oncotator (http://www.broadinstitute.org/cancer/cga/oncotator). The mutational signature analysis in a three-base context is via a NMF algorithm previously developed^23^. To confirm the results, we also used the R package *deconstructSigs* detecting signatures on each sample^55^. The MutSigCV2.0 algorithm was used to identify significantly recurrently mutated genes^24^. Control-FREEC was used to process sequencing reads from tumor and matched normal samples to segmented copy number alterations^29^. The ABSOLUTE algorithm was used to estimate ploidy and purity of tumor samples^30^. The GISTIC 2.0 software was used to identify recurrent copy number alterations^31^. SVs were called by clustering putative breakpoints identified by discordant read pairs and split reads using Manta^44^. Chromothripsis, characterized by dense concentration of SVs in one or few chromosomes, was detected using the set of criteria described in a previous EAC study^20,56^: chromosomes present three times more breaks per Mb than expected and breaks are equally distributed in the genome; chromosomes have evidence of clustering of breakpoints by the Kolmogorov–Smirnov test; chromosomes have at least 10 switches in copy number states. A remarkable phenomenon of localized hypermutation, kataegis, was detected using the R package SeqKat^57^, which defined microclusters of kataegis as clusters that contain at least 4 consecutive SNVs with inter-variant distance less than 2 kb and have hyperscore >5. To detect another major catastrophic event, BFB cycles, we first searched for evidence of clustering of breakpoints, and further reviewed those chromosomes to see if they included loss of telomeric region with neighboring highly amplified region^20^.

### Statistical analysis and clustering of tumor samples

Wilcoxon rank sum tests were used to compare the mutation burden and the proportion of a particular mutation type between Chinese EAC samples and US/UK EAC samples. Fisher's exact tests were used to compare the proportion of chromothripsis and BFB between Chinese EAC samples and US EAC samples. Hierarchical clustering was performed based on SCNA from the combined Chinese and US EAC samples.

## Electronic supplementary material

Supplementary Information

Supplementary Data 1

Description of Additional Supplementary Files

## Data Availability

Binary sequence alignment/map (BAM) files and vcf files have been deposited to the database of Genotypes and Phenotypes (dbGaP). The dbGaP accession assigned to this study is phs001696.v1.p1.
