# Supplementary material for: Whole-genome sequencing of esophageal adenocarcinoma in Chinese patients reveals distinct mutational signatures and genomic alterations
Source: Commun Biol. 2018 Oct 24;1:174. doi: 10.1038/s42003-018-0182-8 (PMC6200836; doi:10.1038/s42003-018-0182-8)
Supplement: Supplementary file 1 — Supplementary Information [file 42003_2018_182_MOESM1_ESM.pdf]

A.

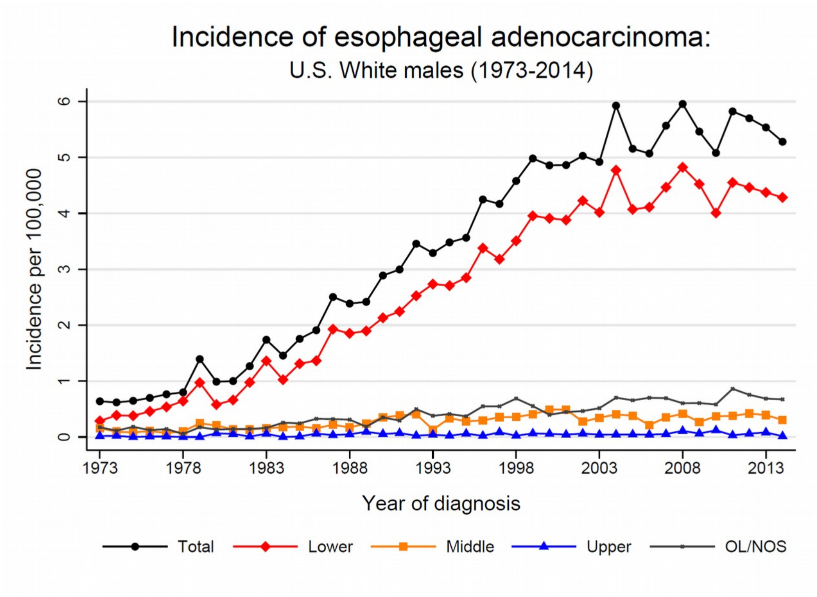

B.

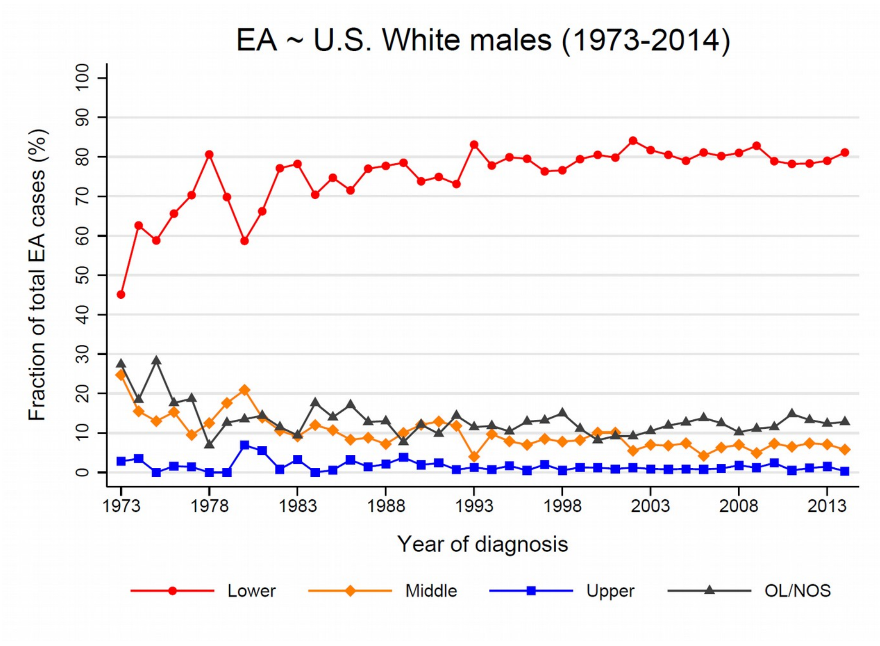

Supplementary Figure 1. **(A)** Incidence of esophageal adenocarcinoma (C15.0-C15.9: 8140/3) among U.S. White males based on SEER9 registry data (1973-2014). Cases coded as 'cervical' (C15.0), 'thoracic' (C15.1), or 'abdominal' (C15.2) were re-classified as 'upper' (C15.3), 'middle' (C15.4), or 'lower' (C15.5), respectively. 'OL/NOS' includes cases coded as 'overlapping' (C15.8) and 'unspecified' (C15.9). **(B)** Distribution of EA cases by anatomic site.

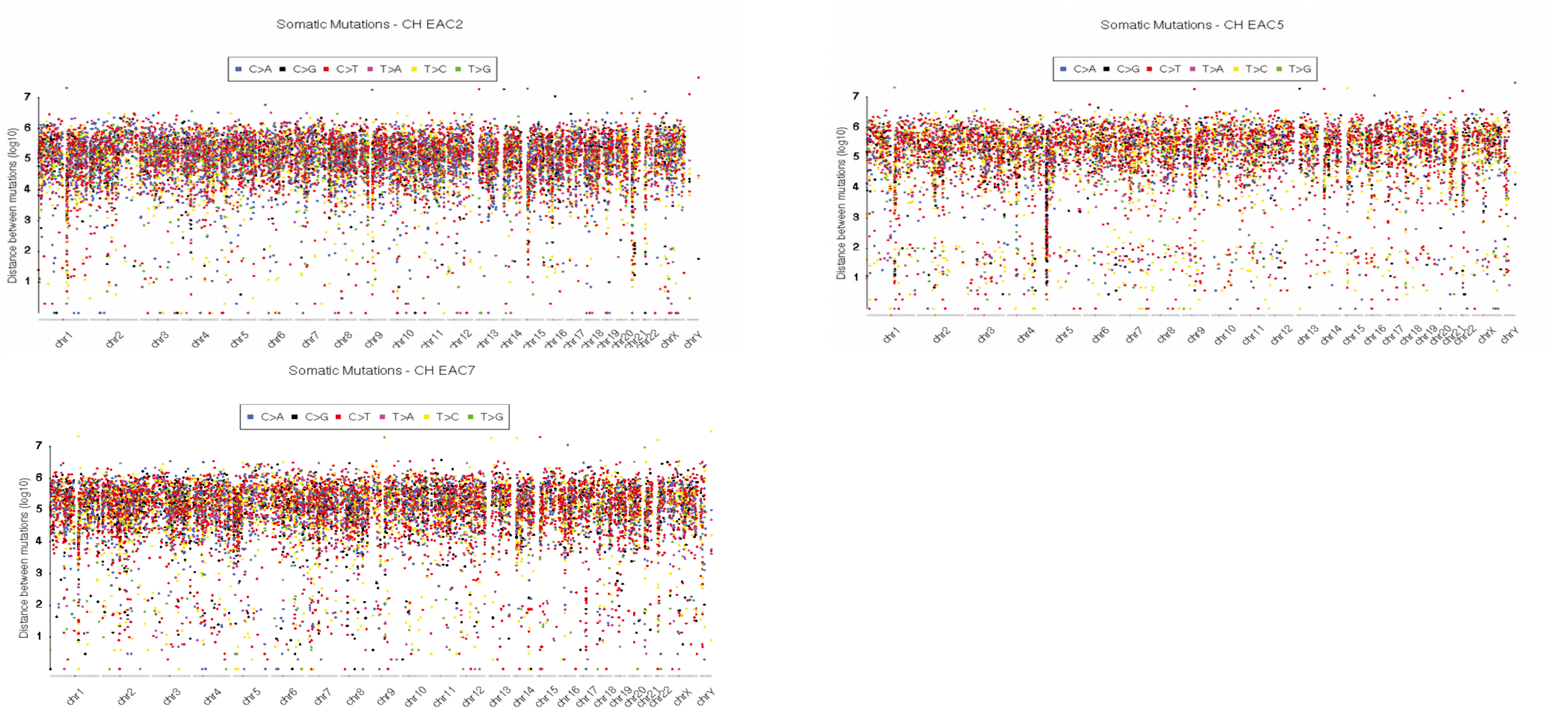

Supplementary Figure 2. Kataegis rainfall plots for Chinese EAC samples. Each of these ‘rainfall’ plots represents an individual tumor in which each dot represents a single somatic mutation ordered on the horizontal axis according to its position in the human genome. The vertical axis denotes the genomic distance of each mutation from the previous mutation.

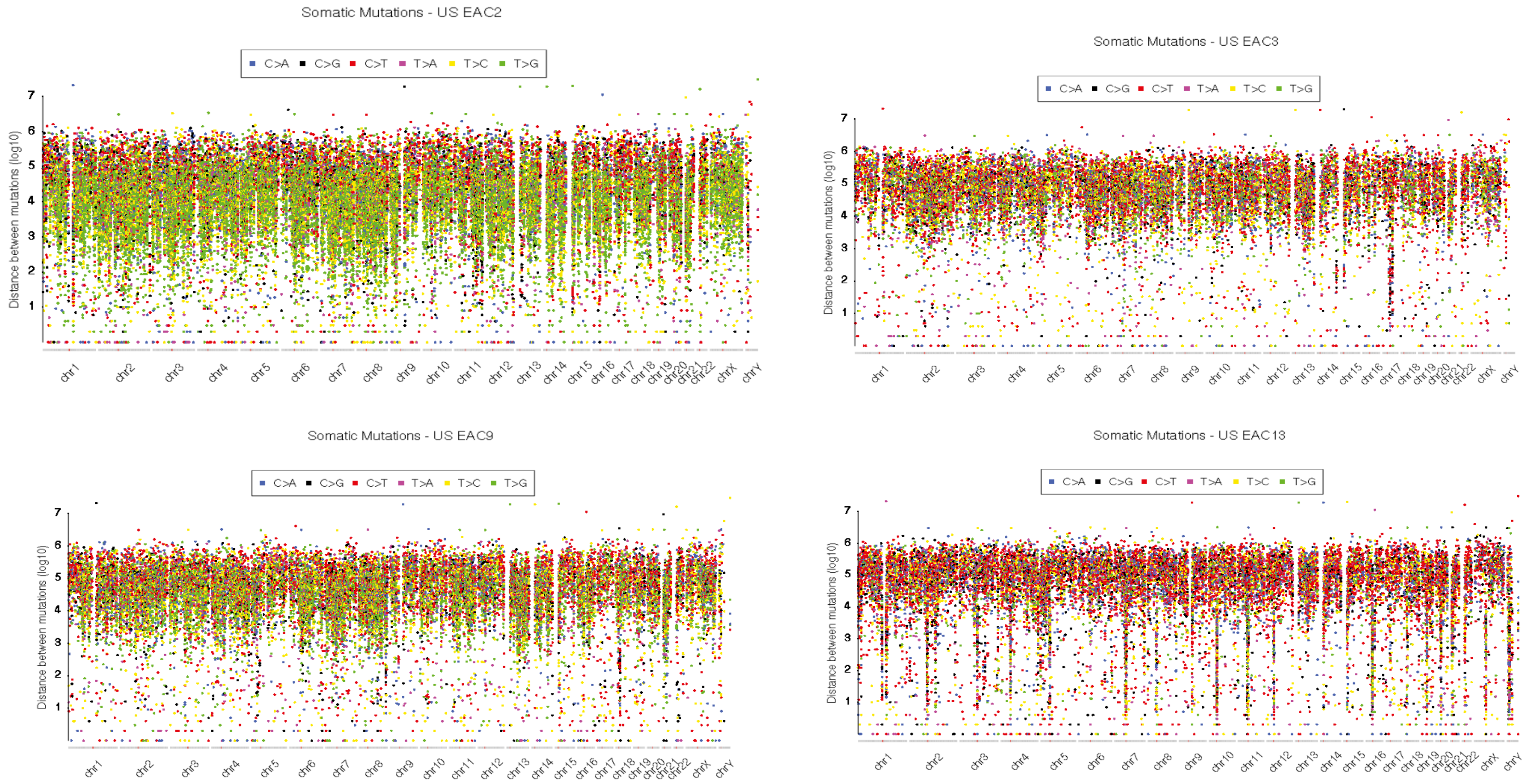

Supplementary Figure 3. Example Kataegis rainfall plots for four US EAC samples. Each of these 'rainfall' plots represents an individual tumor in which each dot represents a single somatic mutation ordered on the horizontal axis according to its position in the human genome. The vertical axis denotes the genomic distance of each mutation from the previous mutation.

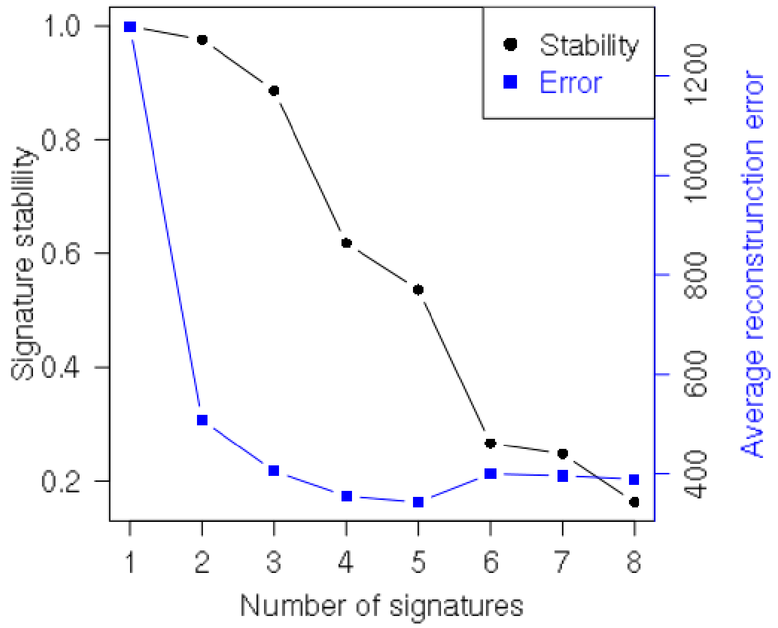

Supplementary Figure 4. signature stability and reconstruction error. Parameters are estimated by the NMF methodology for different numbers of signatures(1-8) explaining the data. Three signatures give the optimal trade-off between signature stability and reconstruction error and were thus selected as the more likely configuration that may explain the observed data

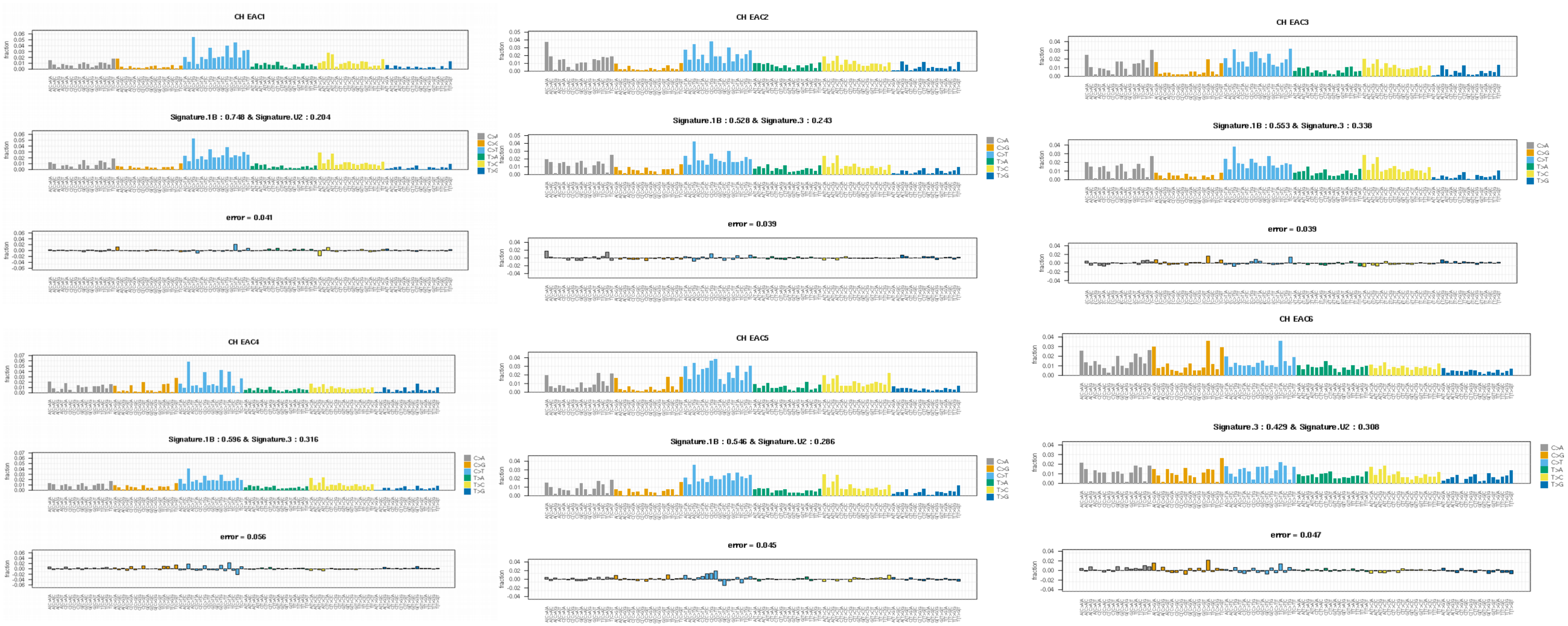

Supplementary Figure 5. Examples of signature identified by deconstructSigs. For each tumor, plot the trinucleotide frequency on the top panel, the resconstructed one on the middle panel, and the difference between the two on the bottom panel.

A

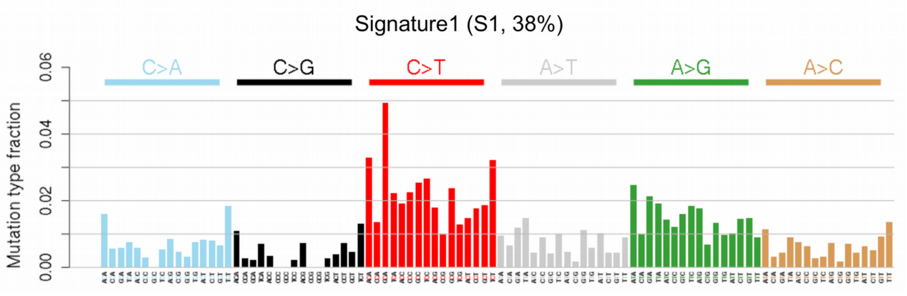

B

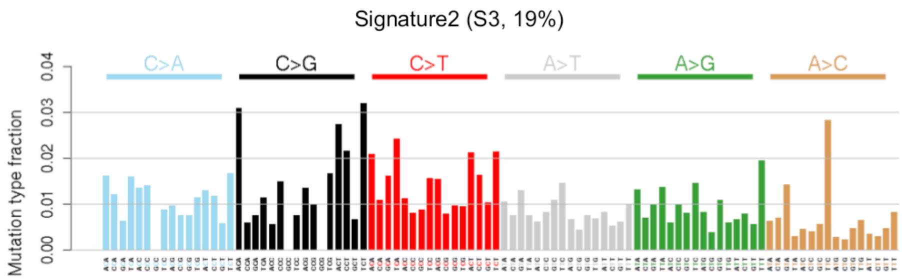

C

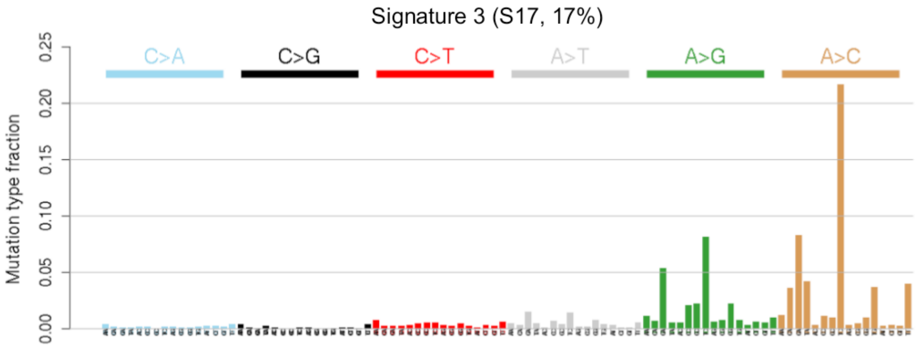

D

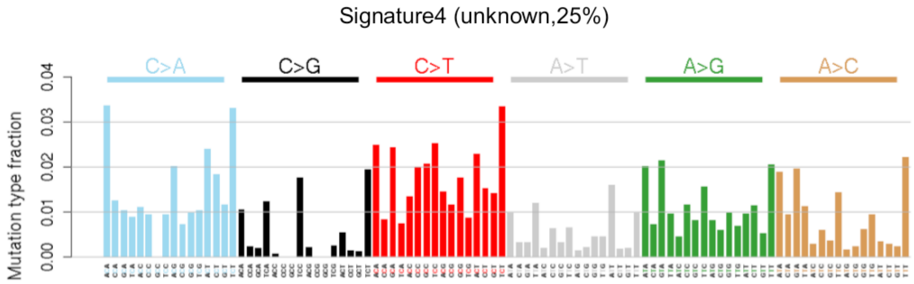

Supplementary Figure 6. Mutational signatures detected in US samples

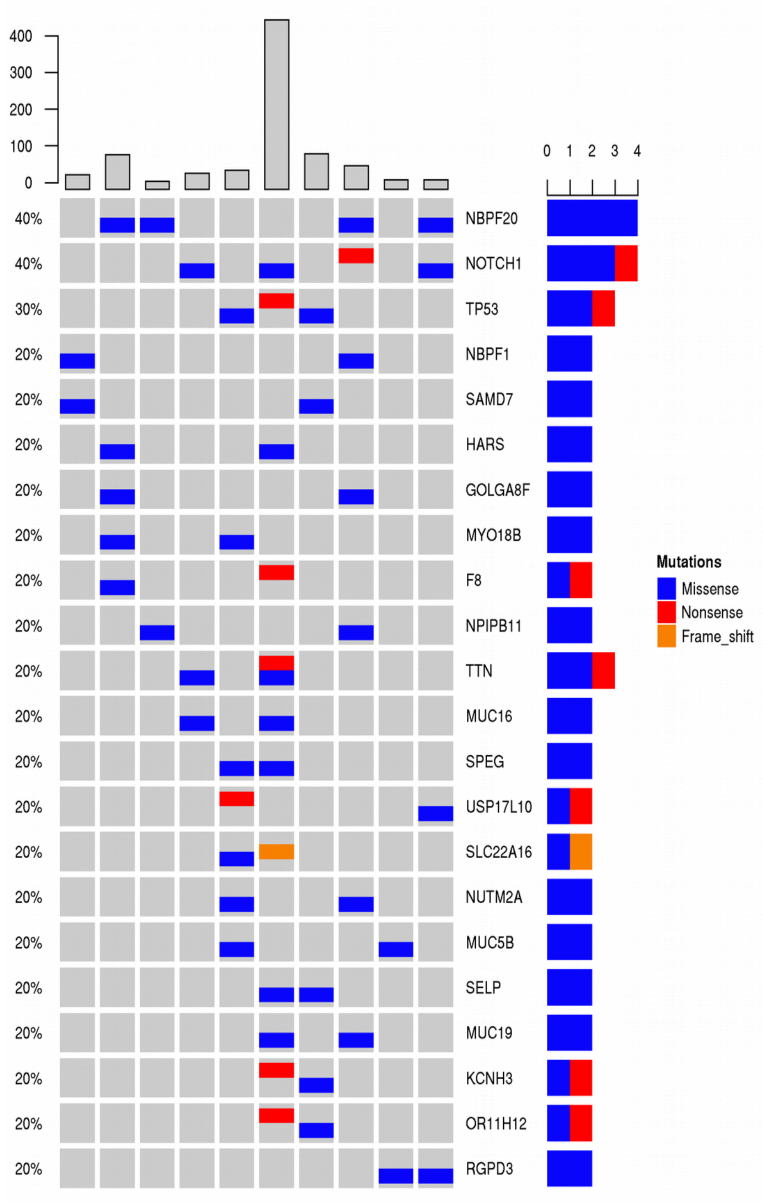

Supplementary Figure 7. The recurrent genes that have nonsilent mutations in Chinese EAC. The bar on the top shows the total number of nonsilent mutations in the genome. The bar on the right shows the number of cancer samples having mutations in a gene.

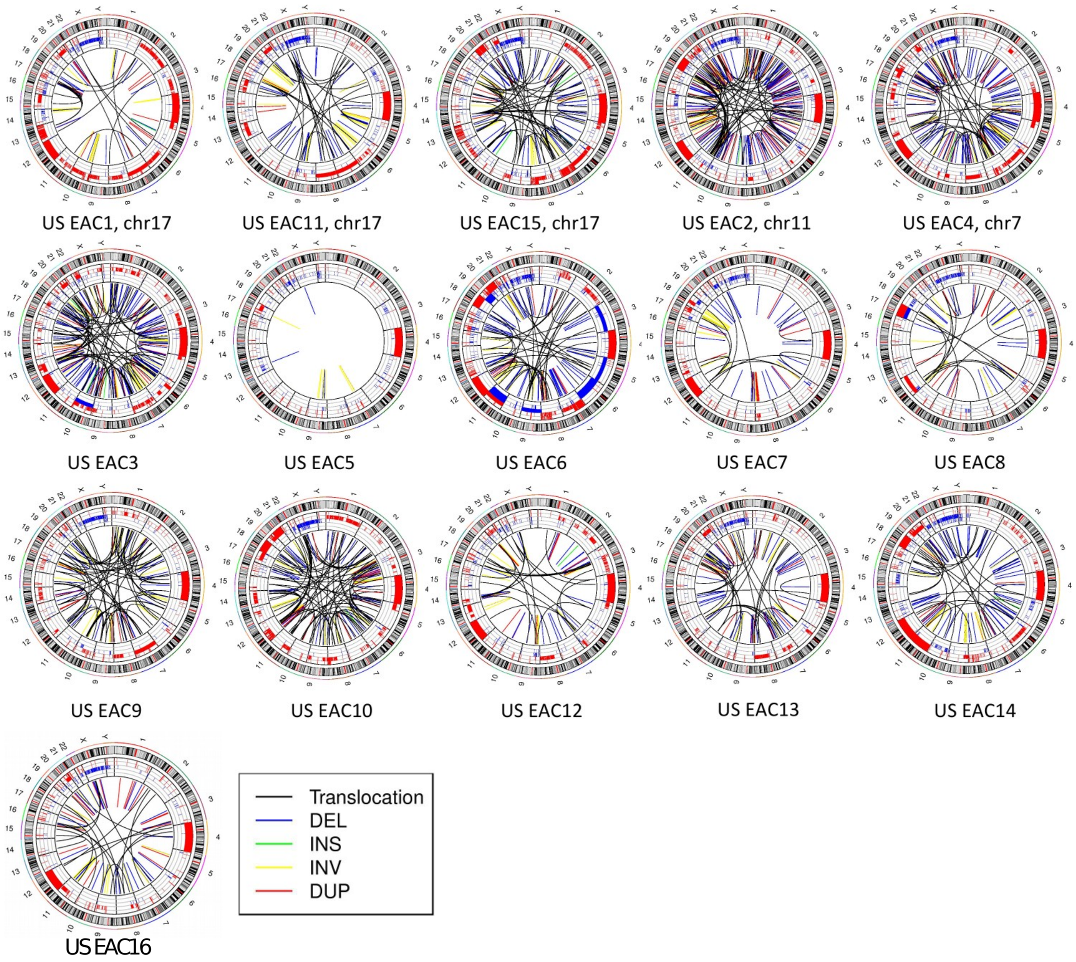

Supplementary Figure 8. Evidence of chromothripsis in 16 US EAC tumors, shown by the circos plots. The outer rings show the copy number alterations (blue represents deletions and red represents amplifications). The inner ring show somatic structural variants (SVs). Color of the lines represents SV types as indicated in the legend. The top 5 EAC samples have evidence of chromothripsis with specific chromosome labeled.

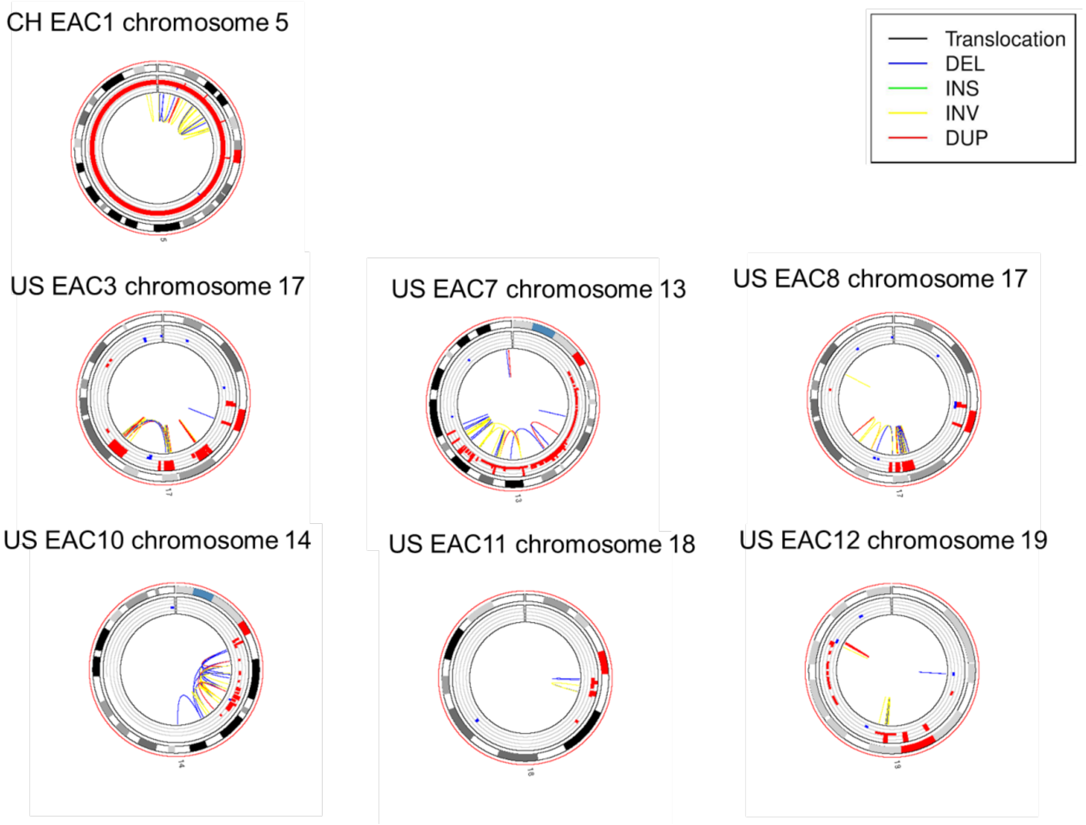

Supplementary Figure 9. Breakage-fusion-bride cycles detected in 1 Chinese EAC sample and 6 US EAC samples. For each sample, the chromosome with BFB events was shown in a CIRCOS plot. The outer rings show the copy number alterations (blue represents deletions and red represents amplifications). The inner ring show somatic structural variants (SVs). Color of the lines represents SV types as indicated in the legend.

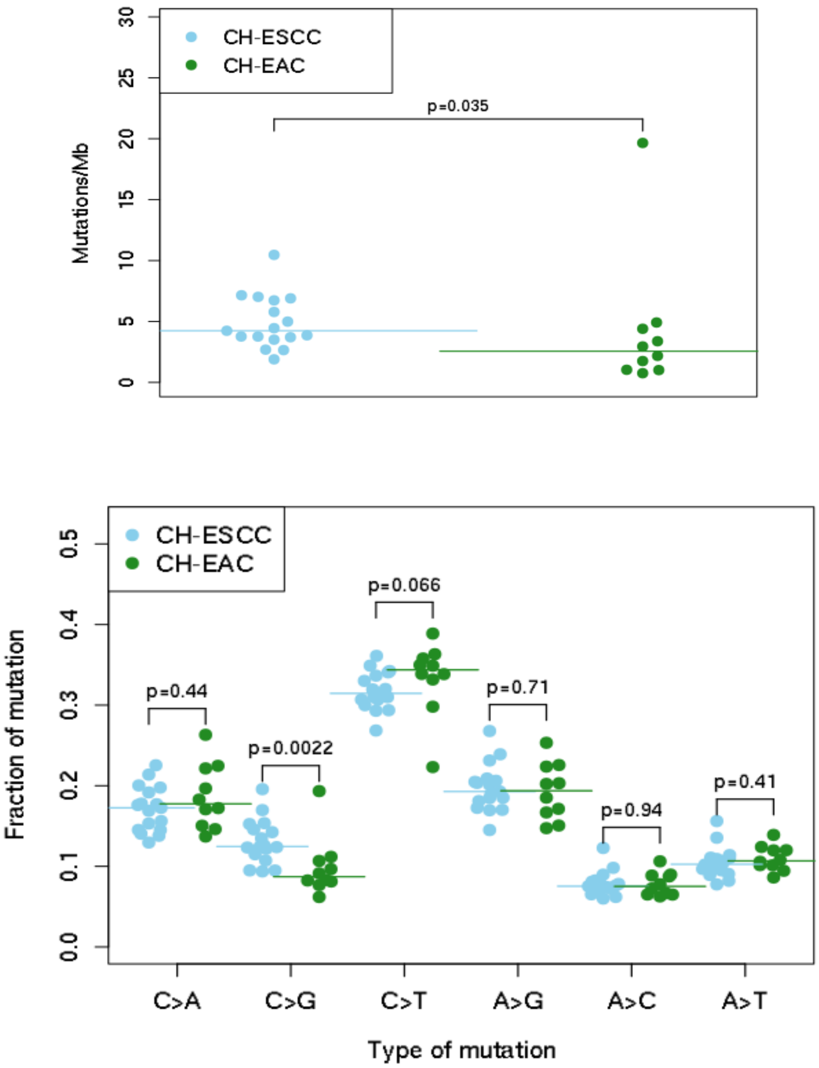

Supplementary Figure 10. Comparison of Chinese ESCC and Chinese EAC on mutation

Supplementary Table 1: Patient and tumor characteristics

Number of patients	10(100%)
Gender	
Female	4(40%)
Male	6(60%)
Age, mean, years (STD)	58.3 (9.8)
Location of tumor	
Lower thoracic esophagus	3(30%)
Middle thoracic esophagus	7(70%)
Tumor stage	
Ⅰ	3(30%)
Ⅱ	4(40%)
Ⅲ	2(20%)
Ⅳ	1(10%)

Supplementary Table 2: List of kataegis detected in Chinese EAC and US EAC

sampleID	chromosome	start	end	nummutations
CH EAC2	21	22615239	22616212	10
CH EAC5	5	2718090	2721130	9
CH EAC5	5	4237197	4238693	10
CH EAC5	5	4450301	4451630	10
CH EAC7	17	37728494	37729577	13
US EAC2	10	42653373	42657716	9
US EAC2	11	110173867	110177356	10
US EAC2	11	112638254	112641958	11
US EAC2	11	132261161	132266294	18
US EAC2	13	27168678	27173115	17
US EAC3	14	100164775	100166375	10
US EAC3	17	38944923	38947390	14
US EAC3	17	50941996	50949101	26
US EAC4	8	80003439	80005227	9
US EAC6	12	25984651	25986014	10
US EAC7	13	73580723	73584258	12
US EAC7	13	76867309	76868921	11
US EAC7	18	21518674	21523961	28
US EAC8	17	37913283	37915526	16
US EAC8	17	49458626	49461712	18
US EAC9	18	21559848	21561840	13
US EAC9	18	22878478	22881969	18
US EAC10	1	187384561	187388229	10
US EAC10	1	194257787	194261364	11

US EAC10	23	53700566	53701934	10
US EAC10	23	53858238	53860137	14
US EAC12	1	166844901	166845835	13
US EAC12	18	12509044	12511044	9
US EAC13	1	121376770	121382358	15
US EAC13	10	39117715	39130355	45
US EAC13	10	42814052	42818010	17
US EAC13	11	50712442	50719237	14
US EAC13	11	51572063	51578768	12
US EAC13	12	34833465	34840234	10
US EAC13	16	33864779	33872628	29
US EAC13	16	34175440	34179715	11
US EAC13	16	35223939	35232042	16
US EAC13	18	18511561	18520313	19
US EAC13	19	27735954	27739271	9
US EAC13	2	89865646	89879245	49
US EAC13	2	132994334	132998365	10
US EAC13	23	58559813	58565050	9
US EAC13	3	121201629	121202643	9
US EAC13	3	130488685	130492497	15
US EAC13	4	49648820	49656970	25
US EAC13	7	57546111	57550972	21
US EAC13	7	57943228	57950793	16
US EAC13	7	61079188	61083693	12
US EAC13	8	43787659	43794772	17
US EAC15	16	46495992	46496533	4
US EAC16	11	130734348	130737719	22
US EAC16	11	131538035	131540740	14

Supplementary Table 3:Tumor ploidy and purity

Sample	Purity	Ploidy
CH EAC1	0.76	1.59
CH EAC2	0.74	2.03
CH EAC3	0.83	1.88
CH EAC4	0.67	2.45
CH EAC5	0.78	1.93
CH EAC6	0.83	1.96
CH EAC7	0.74	2.06
CH EAC8	0.85	1.93
CH EAC9	0.87	1.98
CH EAC10	0.75	2.09
