# Supplementary material for: Whole-genome sequencing of esophageal adenocarcinoma in Chinese patients reveals distinct mutational signatures and genomic alterations
Source: Commun Biol. 2018 Oct 24;1:174. doi: 10.1038/s42003-018-0182-8 (PMC6200836; doi:10.1038/s42003-018-0182-8)
Supplement: Supplementary file 3 — Description of Additional Supplementary Files [file 42003_2018_182_MOESM3_ESM.pdf]

Description of Additional Supplementary Files

File Name: Supplementary Data 1

Description: The supplementary data file contains the recurrent somatic copy number alterations (SCNA) for Chinese and US esophageal adenocarcinoma, detected by the GISTIC software.
